# Ergot Alkaloids Affect Foraging Activity of the Slime Mold *Physarum polycephalum*

**DOI:** 10.3390/toxins18070282

**Published:** 2026-06-27

**Authors:** Jordan R. Sexstone, Abigail M. Jones, Daniel G. Panaccione

**Affiliations:** School of Natural Resources and the Environment, West Virginia University, Morgantown, WV 26506, USA; jrs00065@mix.wvu.edu (J.R.S.); amj0043@mix.wvu.edu (A.M.J.)

**Keywords:** ergot alkaloids, *Aspergillus*, *Physarum*, lysergic acid amides, microbial interactions

## Abstract

We tested whether ergot alkaloids would affect the interaction of a protist with an ergot alkaloid-producing fungus by studying the response of the plasmodial slime mold *Physarum polycephalum* to cultures of *Aspergillus leporis*. Ergot alkaloid profiles were manipulated by culturing *A. leporis* and its *easD* knockout derivative on media for 6 or 13 days. Six-day-old, wild-type *A. leporis* samples contained abundant lysergic acid α-hydroxyethylamide (LAH) that was greatly depleted by 13 days. The intermediate chanoclavine-I was the predominant ergot alkaloid in all samples of the *easD* knockout. Inoculum of *P. polycephalum* was placed equidistant between the fungus-colonized agar medium explant, and the preference of the slime mold for either fungal explant was observed. When offered 6-day-old samples of wild-type *A. leporis* (containing mainly LAH) or the *easD* knockout (containing mainly chanoclavine-I), plasmodia of *P. polycephalum* preferred the *easD* knockout strain over wild type by a significant margin (*p* = 0.0002). When given the same options at 13 days, there was no longer a preference for the *easD* knockout over wild type, which by that time had lost more than 90% of its LAH. Our data demonstrate that ergot alkaloids, including LAH and chanoclavine-I, affect the interaction of *P. polycephalum* with *A. leporis*.

## 1. Introduction

Ergot alkaloids are a family of specialized metabolites produced by several lineages of fungi [1,2,3]. Diverse metabolites from different branches of the ergot alkaloid pathway affect organisms from multiple kingdoms, but ergot alkaloids are best known for their effects on humans and livestock mammals. Consumption of ergot alkaloid-contaminated rye and other grains caused periodic mass poisonings of people historically, but diversification of diets and grain-cleaning practices have almost eliminated that issue [1,2,4]. Ergot alkaloids still affect humankind, however, because their ability to act as agonists or antagonists of different neurotransmitters has led to their use in numerous pharmaceuticals [1,5,6]. Ergot alkaloids produced by fungal symbionts of forage grasses are a concern in agriculture, where they cause significant problems with health and fertility in grazing animals [2,7]. Although not as intensively studied, ergot alkaloids in several fungi also adversely affect insects [8,9,10,11,12], nematodes [3,13], and bacteria [3,14]. Studies of the potential effects of ergot alkaloids, or any mycotoxin, on protists are notably lacking in the published literature. This knowledge gap is surprising considering that many mycotoxin producers are likely to live in habitats colonized by protozoans, including slime molds of the class Myxogastria that engulf fungal spores and mycelial fragments as part of their diets [15,16,17].

**Figure 1:**
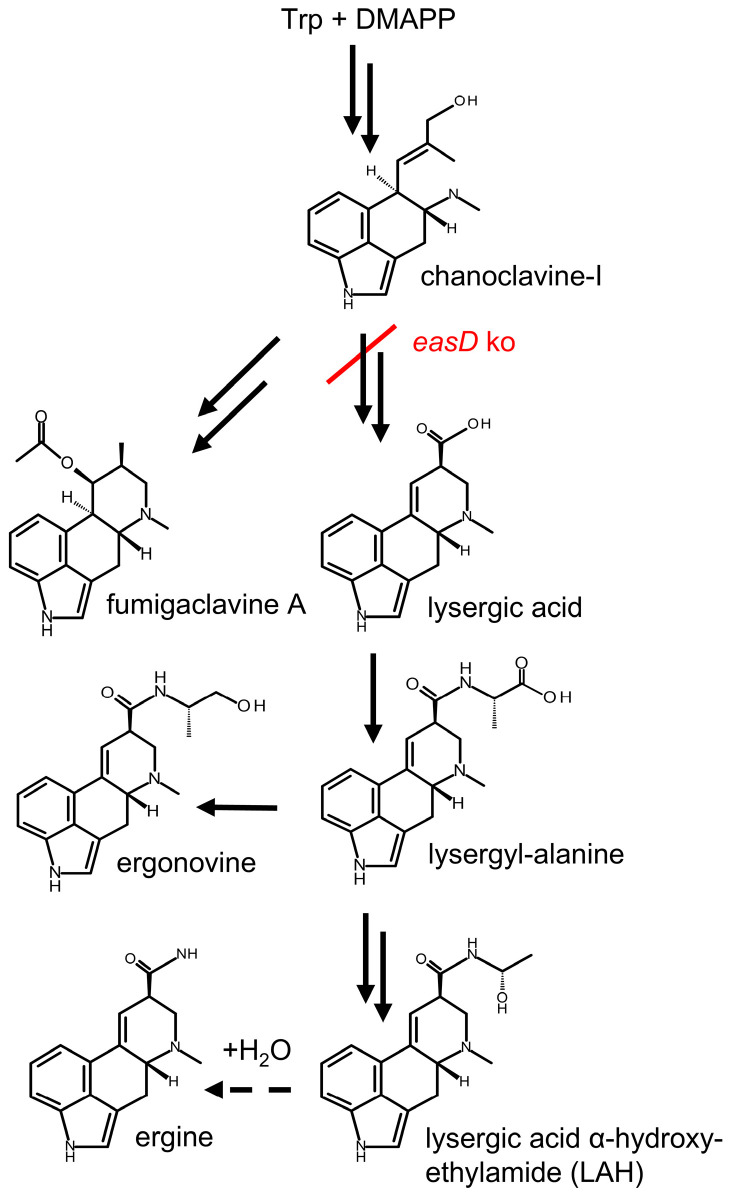
Pathway from tryptophan (Trp) and dimethylallylpyrophosphate (DMAPP) to ergot alkaloids of the lysergic acid amide branch and the fumigaclavine A branch. The step at which the enzyme encoded by *easD* acts is indicated in red. Multiple arrows indicate omitted intermediate steps. The branch to fumigaclavine A contains a second copy of *easD* that is active in older cultures and can supplement biosynthesis in both branches [29].

Although ergot alkaloids originally gained notoriety because of their accumulation in plants eaten by people and livestock [1,2,4], they have recently been found as major products of several soil-inhabiting fungi, too. Among these soil-inhabiting ergot alkaloid producers are numerous *Metarhizium* species [18] that grow in soil and as plant symbionts but also parastize insects [19]. Several additional ergot alkaloid producers have been discovered among dung and soil-inhabiting members of the diverse fungal genus *Aspergillus*. *Aspergillus leporis* was originally found in rabbit dung [20] but later isolated from soils in the western United States [21,22]. *Aspergillus homomorphus* was discovered in soil near the Dead Sea [23] and has also been found in stored millet grain [24]. *Aspergillus hancockii* was isolated and characterized from agricultural soils in Australia [25], and *A. aspearensis* was described from soil from Iran [26]. Each of these *Aspergillus* species, as well as the *Metarhizium* species, makes ergot alkaloids of the lysergic acid amide class, with lysergic acid α-hydroxyethylamide (LAH) as the primary pathway product [18,27,28] (Figure 1). Whereas this pathway branch has been documented in at least two plant-inhabiting fungi [2], its commonality with numerous soil-inhabiting fungi from two diverse lineages is intriguing.

Considering the apparent overlap in niches of slime molds with ergot alkaloid-producing fungi and the broad spectrum of activities of these chemicals against a range of potential competitors, we hypothesized that slime molds would be sensitive to ergot alkaloids, particularly those from the pathway branch common to soil-inhabiting fungi. To test this hypothesis, we investigated the interaction of the plasmodial slime mold *Physarum polycephalum* with the ergot alkaloid-producing, soil-inhabiting fungus *Aspergillus leporis*. *Physarum polycephalum* was a logical choice for the slime mold in this study because of the ease with which it can be cultured, its well-documented decision-making abilities [30,31], and the fact that fungi constitute some component of its diet [15,16,17]. In fact, Stephenson and Stempen [16] have speculated that *P. polycephalum* “can probably be regarded as primarily fungivorous.” We chose the fungus *Aspergillus leporis* as the ergot alkaloid producer for this study for the following reasons: it contains the lysergic acid amide branch of the pathway common to soil-inhabiting ergot alkaloid producers [27]; it produces high concentrations of ergot alkaloids in vitro while secreting much of them into the surrounding medium, facilitating diffusion [27]; and, we have a mutant strain of it that is deficient in lysergic acid amides [9]. We used the natural temporal progression of ergot alkaloid accumulation and turnover documented by Jones et al. [27], and the lysergic acid amide-deficient mutant knocked out at the *easD* locus [9] to assemble samples that varied qualitatively and quantitatively in ergot alkaloids, and then studied the response of plasmodia of *P. polycephalum* to these samples in paired preference tests.

## 2. Results

### 2.1. Manipulation of Ergot Alkaloid Profiles by Modifying Strain and Culture Time

**Figure 2:**
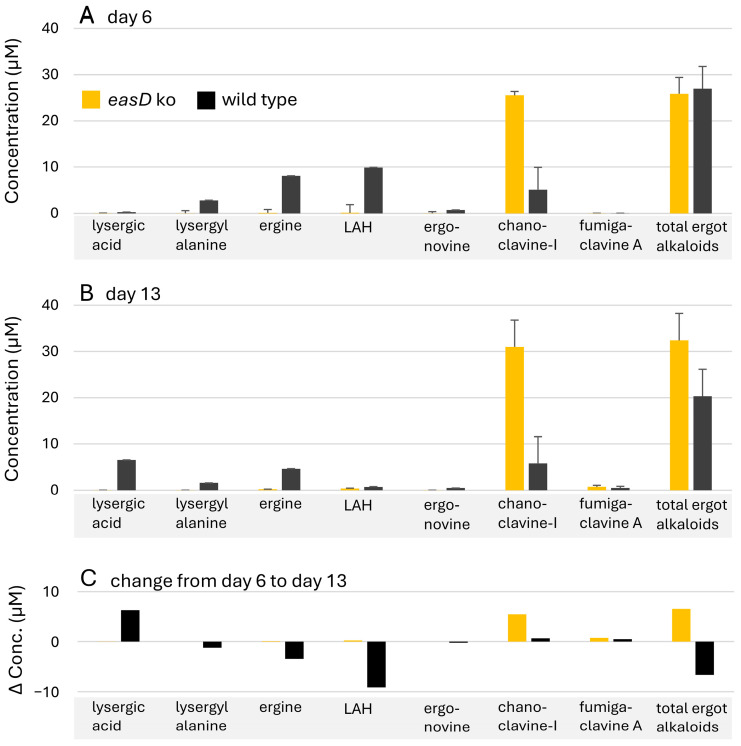
Concentrations of ergot alkaloids in (**A**) 6-day-old cultures, (**B**) 13-day-old cultures, and (**C**) the change in concentration from day 6 to day 13. For panel (**A**), values represent overall means from eight trials of three replicates each of SYE samples colonized for six days by wild-type *A. leporis* (black bars) or its *easD* knockout derivative (yellow bars). Panel (**B**) represents an overall mean for 6 trials of three replicate samples of the same two fungal strains. Error bars (which are present on all bars in panels (**A**,**B**), though small on some) represent the standard error of the mean. Panel (**C**) lacks error bars because the values represent the difference in the means of the 13-day samples for each alkaloid minus the mean of the corresponding 6-day samples, and thus are a single value; variation inherent to these concentration values is reflected by the error bars in panels (**A**,**B**). Chanoclavine-I, ergonovine, and fumigaclavine A were quantified relative to authentic standards. Lysergic acid, lysergyl-alanine, LAH, and ergine were quantified relative to an ergonovine standard with the same fluorophore, so values for those alkaloids must be considered as relative to ergonovine as opposed to absolute.

Samples with varied concentrations of ergot alkaloids were obtained by growing wild-type *A. leporis* and a previously described *easD* knockout strain [9] on sucrose-yeast extract agar (SYE) for 6 or 13 days. The *easD* knockout strain had its functional copy of the *easD* gene from the lysergic acid amide biosynthetic synthesis cluster mutated by a CRISPR/Cas9-based approach, resulting in a significant reduction in the primary pathway product LAH (*p* < 0.001) when alkaloids were measured by high-performance liquid chromatography (HPLC) at 6 days after inoculation (Figure 2). This knockout mutation also led to a significant increase in chanoclavine-I (*p* < 0.001), the early pathway intermediate that is the substrate for EasD (Figure 1), such that chanoclavine-I dominated the ergot alkaloid profile of the knockout strain (Figure 2). A second copy of *easD*, found in a satellite gene cluster that encodes genes capable of converting chanoclavine-I into fumigaclavine A [29], allowed minimal accumulation of LAH in some cultures of the knockout mutant as well as accumulation of some fumigaclavine A, particularly in 13-day-old cultures (Figure 2). When assessed by HPLC at 13 days after inoculation, the previously high concentrations of LAH in the wild type were reduced by over 90% relative to values at day 6 (Figure 2), as documented previously in a reproducible progression in alkaloid changes in this fungus over time [27]. At the same time, the concentration of chanoclavine-I in the *easD* knockout strain increased further. Despite these distinct changes in ergot alkaloid profiles, the total (summed) quantities of ergot alkaloids in the wild type compared to the knockout strain did not differ significantly on day 6 (*p* = 0.64). On day 13, the concentrations of total ergot alkaloids in the wild type versus the knockout were also not significantly different (*p* = 0.09), though this *p* value may be affected by the relatively high variation in chanoclavine-I accumulation.

### 2.2. Effect of Ergot Alkaloid-Producing Fungi on Foraging Preferences of Plasmodia of Physarum polycephalum

**Figure 3:**
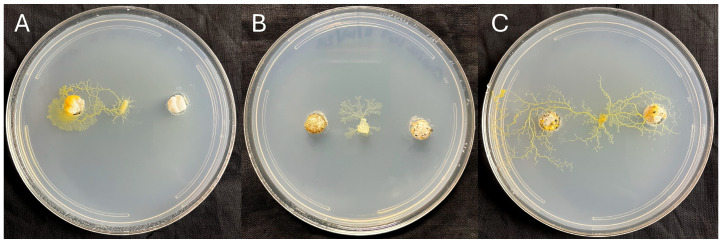
Representative outcomes of preference trials with fungus-colonized SYE explants and plasmodia of *P. polycephalum*. (**A**) The plasmodium chose an explant colonized by the *easD* knockout of *A. leporis* for 6 days over one colonized by the wild type of *A. leporis*. (**B**) The plasmodium was scored as undecided because it contacted neither fungus-colonized explant. (**C**) The plasmodium was scored as undecided because it contacted both fungus-colonized explants. Explants in panels (**B**,**C**) are from 13-day-old cultures of *A. leporis* and contain some sclerotia of the fungus visible as small black spheres on the fungus-colonized agar cores.

Explants of fungus-colonized SYE cultures of wild-type *A. leporis* and its *easD* knockout derivative containing measured concentrations of different ergot alkaloids (as described above) were offered to plasmodia of *P. polycephalum* in paired preference tests. After 16 h, plasmodia were recorded as preferring one or the other explant if it was contacting that explant (Figure 3). Plasmodia were scored as undecided if they contacted neither nor both explants. In a retrospective survey of photographs taken of several preference trials, plasmodia contacted neither of the explants in 21 of 23 assays scored as undecided. These data indicate that the vast majority of the undecided plasmodia rejected both offerings of fungus-colonized SYE.

**Figure 4:**
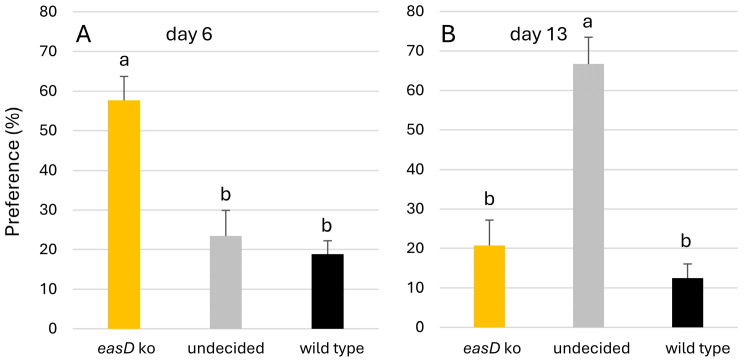
Preference of plasmodia for SYE colonized by the *easD* knockout strain of *A. leporis* (yellow bars) compared to medium colonized by the wild-type strain of *A. leporis* (black bar). Plasmodia scored as undecided are indicated with gray bars. (**A**) Comparisons of paired explants of the indicated fungal strains grown on SYE for 6 days. (**B**) Data from SYE explants colonized by the indicated fungal strains for 13 days. Error bars represent the standard error of the mean. Means marked with different letters within each panel differ significantly (*p* < 0.05) in a Tukey’s honestly significant difference test.

When presented with 6-day-old samples of SYE colonized with wild-type *A. leporis* (with LAH as the most abundant ergot alkaloid) or the *easD* knockout of *A. leporis* (containing mainly chanoclavine-I), plasmodia of *P. polycephalum* preferred the *easD* knockout strain over the wild type by a significant margin (*p* = 0.0002) (Figure 4A). The remaining plasmodia were divided approximately equally between those that were undecided and those that chose the wild-type-colonized SYE. When given analogous options cored from 13-day-old cultures, the most common outcome was for plasmodia of *P. polycephalum* to not make a clear choice (*p* = 0.0001) (Figure 4B). With these older samples, there was no preference for *easD* knockout over wild type (*p* = 0.57), which by 13 days had lost more than 90% of its LAH (Figure 2).

**Figure 5:**
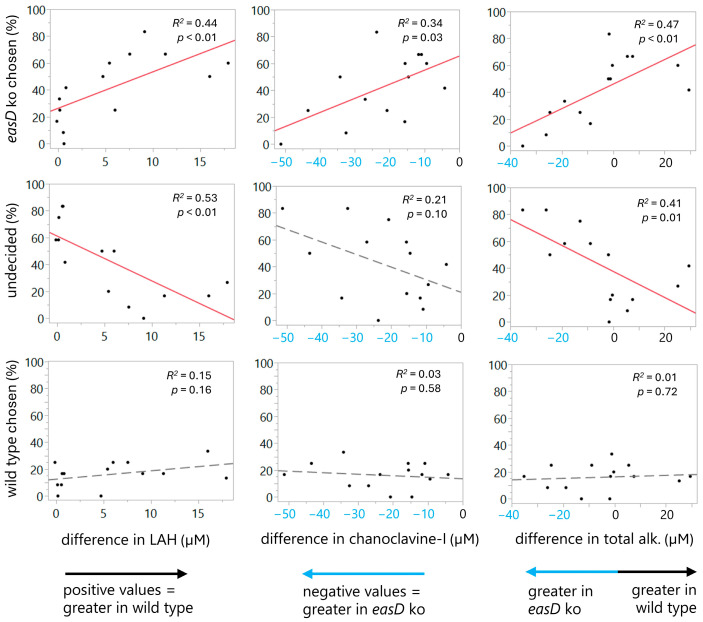
Correlations of preference of plasmodia of *P. polycephalum* relative to differences in concentrations of ergot alkaloids lysergic acid α-hydroxyethylamide (LAH), chanoclavine-I, or total ergot alkaloids in paired samples from each of the 14 paired trials (eight at day 6 and six at day 13). Each dot represents data from a trial in which alkaloid concentration was determined and for which choice values also were documented. Red lines indicate correlations with *p* values < 0.05, and dashed gray lines represent correlations with *p* values > 0.05. Alkaloid values on the x-axis are the differences in means of the values measured for explants colonized by wild-type *A. leporis* minus those measured for the *easD* knockout explant with which it was paired. For this reason, negative values on the x-axis (shown in blue) result when the value for the *easD* knockout sample was greater than that measured in the wild-type sample with which it was paired.

We combined the preference data from 6-day trials and 13-day trials with alkaloid concentration data collected from triplicate explants from the same culture plates and correlated explant choice with differences in concentration of the major alkaloids, varying in concentration in those pairings, LAH and chanoclavine-I. When differences in LAH were high between paired samples, many plasmodia chose SYE colonized by the *easD* knockout (Figure 5). When the difference in LAH concentration was low between paired samples (which occurred most often in 13-day samples), then many plasmodia were undecided. Plasmodial choices indicated an inverse relationship with chanoclavine-I relative to the selection of the *easD* knockout. In test pairings where there was a large difference in the concentrations of chanoclavine-I, plasmodia tended to be undecided (Figure 5). When paired samples had a smaller difference in chanoclavine-I concentration, plasmodia were more likely to choose the *easD* knockout. When plasmodial preference was compared across differences in total concentration of ergot alkaloids, *P. polycephalum* was mainly undecided when total ergot alkaloid concentration was higher in the *easD* knockout than in the wild type, and when there was no difference in concentration, or when the wild type had a greater concentration, then plasmodia of *P. polycephalum* chose the *easD* knockout-colonized SYE explant more frequently (Figure 5). Interestingly, the choice of wild-type-colonized SYE was made at a low level across pairings and did not appear to correlate with concentrations of the ergot alkaloids. Tests of correlations with other ergot alkaloids, which were found in lower concentrations, yielded some marginal correlations with the other lysergic acid amides (lysergyl-alanine, ergine, and ergonovine) (Table S1); the difference in concentration in each of these lysergic acid amides correlated with the difference in concentration of LAH (Table S2).

**Figure 6:**
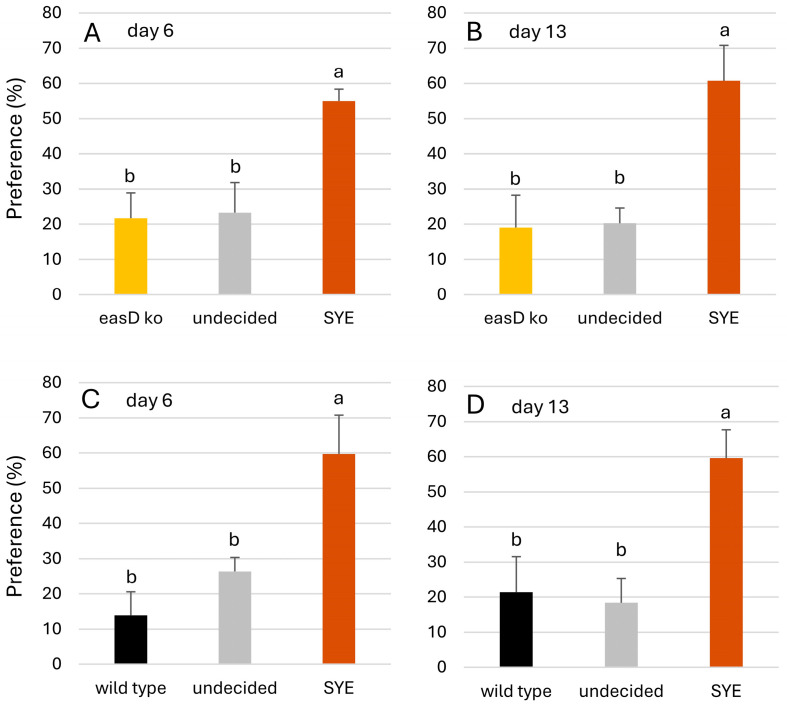
Results of preference tests in which noncolonized sucrose-yeast extract agar (SYE; red bars) was paired with SYE colonized by the *easD* knockout strain of *A. leporis* (yellow bars; (**A**,**B**)) or SYE colonized by the wild-type strain of *A. leporis* (black bars; (**C**,**D**)). Fungus-colonized explants in (**A**,**C**) were grown for 6 days, whereas those in (**B**,**D**) were grown for 13 days. Error bars represent the standard error of the mean. Means marked with different letters within each panel differ significantly (*p* < 0.05) in a Tukey’s honestly significant difference test.

In additional preference tests, including presumably higher quality, noncolonized SYE explants versus those colonized by either wild-type *A. leporis* or the *easD* knockout strain, plasmodia chose the noncolonized SYE sample most of the time (*p* < 0.05) but still chose the fungus-colonized SYE in 14% to 22% of the trials (Figure 6). These data may represent a baseline level of random selection of the lower-quality food options in the preference test as we constructed it. The values of 14% to 22% align well with the 12% to 19% of tests in which *P. polycephalum* plasmodia selected wild-type *A. leporis*-colonized SYE when it was paired with the *easD* knockout (Figure 4) and during which preference for the wild type did not correlate well with differences in concentrations of the major ergot alkaloids (Figure 5).

## 3. Discussion

### 3.1. Factors Affecting the Preference of Plasmodia for Different Sample Explants

When presented with samples of fungus-colonized agar medium differing primarily in the species of ergot alkaloids present, plasmodia of *P. polycephalum* migrated toward and interacted with the samples in a nonrandom manner. Cores of fungus-colonized medium (and those of noncolonized medium) that had lower concentrations of the ergot alkaloids LAH and chanoclavine-I were preferred.

Our fungus-colonized SYE cores were likely a mixture of attractant (nutritive) and repellent compounds. We note that sucrose (the primary carbon source in SYE) is a poor attractant for *P. polycephalum* [32], but SYE was our preferred medium for this study because of its ability to promote the accumulation of ergot alkaloids in *A. leporis* [9,27,29]. The amino acids in yeast extract, a complex mixture present at 10% wt/vol in SYE, do serve as chemoattractants for plasmodia of *P. polycephalum* [33,34]. In addition, since fungi in general are a foraging option for *P. polycephalum* [15,16,17], the fungus itself may produce chemicals that attract plasmodia. Indirect evidence for chemoattraction of *P. polycephalum* to fungi includes the finding that the volatile organic compound 3-octanone, associated with fungus-like odor, was a chemoattractant to plasmodia [35]. Such attractants may be overridden, of course, by other deterrent fungal metabolites, which our data suggest include certain ergot alkaloids.

Preference for agar cores colonized by the *easD* knockout over those colonized by the wild-type strain of *A. leporis* in the 6-day-old sample trials indicates that LAH, the most abundant ergot alkaloid in the wild type at that time, was a stronger repellent than chanoclavine-I, the predominant ergot alkaloid in the *easD* knockout. Correlation data suggest that when concentrations of chanoclavine-I reached high enough levels in the *easD* knockout, it also repelled plasmodia, which in those tests chose neither explant and were scored as undecided.

The alkaloids that appeared to have the greatest effect on plasmodia of *P. polycephalum* also adversely affected animals in their interactions with ergot alkaloid-producing fungi. LAH contributed to the virulence of *Metarhizium brunneum* [12] and *A. leporis* [9] to the model lepidopteran insect *Galleria mellonella*. Remarkably, the LAH-deficient mutant of *M. brunneum* accumulated other lysergic acid amides (ergonovine and lysergyl-alanine) at high levels, indicating a particular importance for LAH in insect virulence. The *A. leporis* mutant studied for insect virulence was the same chanoclavine-I-accumulating, *easD* knockout strain included here, indicating that chanoclavine-I cannot substitute for LAH in its effects on insects. Chanoclavine-I was among two ergot alkaloids that individually or in combination contributed to feeding deterrence of rabbits (*Oryctolagus cuniculus*) offered perennial ryegrass infected with different mutants of the ergot alkaloid-producing, symbiotic fungus *Epichloë hybrida* [36]. Chanoclavine-I, on the other hand, was not very effective in protecting the perennial ryegrass hosts of mutant *Epichloë* species against black cutworms (*Agrotis ipsilon*) [10] or black beetles (*Heteronychus arator*) [8]; lysergic acid derivatives produced by the respective wild-type fungi were necessary to achieve good protection against these insects [8,10]. These combined studies support the hypothesis that ergot alkaloids that accumulate from different steps along the pathway contribute to different benefits for the fungus that is producing the alkaloids related to self-preservation, resource acquisition, and resource preservation.

### 3.2. Strengths, Limitations, and Other Attributes of the Experimental Approach

Use of an ergot alkoid-producing fungus for these studies, as opposed to isolated chemicals, was necessary because relevant alkaloids, such as LAH and chanoclavine-I, are not available commercially and would be prohibitively difficult to produce and purify on a scale that would allow them to be tested directly in a highly replicated study. The chanoclavine-I used as an analytical standard in our study was acquired in a small quantity in 2012 and is no longer commercially available. We are not aware of LAH ever having been commercially available. Among the ergot alkaloid-producing fungi available to us for this study, *A. leporis* was the best choice for several reasons: it has an ergot alkaloid profile representative of other soil-inhabiting, ergot alkaloid-producing fungi; it produces high concentrations of ergot alkaloids in agar cultures; most of its ergot alkaloids are secreted and diffuse into the culture medium; its alkaloid content can be manipulated by culture age; and, we have a knockout mutant of it that has a very different ergot alkaloid profile from the wild type.

The accumulation of LAH in young cultures of *A. leporis* and its decline in concentration in older cultures is a phenomenon documented previously [27]. LAH, as a hemiaminal, is relatively unstable and spontaneously converts to ergine [37,38]. The lysergic acid that accumulates in older cultures could arise from one or both of two mechanisms: it could be produced by deamination of ergine, and/or it could be an on-pathway intermediate that accumulates as opposed to serving as substrate for the lysergyl peptide synthetase complex responsible for assembling lysergic acid amides.

Our scoring of plasmodia that chose neither sample, and those that chose both samples in a single category, labeled as undecided, may have limited the inferences we could draw about the repellence of certain alkaloids. A choice of neither explant would be a response to approximately equally high repellent conditions, whereas a choice of both would be a response to approximately equally low concentrations of repellents. Our observation that over 90% of the 23 photographed undecided plasmodia had selected neither explant indicated that, in most cases, undecided samples were repelled from both explants.

One interpretation of the data in this study is that the most common decision made by plasmodia of *P. polycephalum* was whether to select the *easD* knockout or remain undecided. The occasional selection of wild-type *A. leporis*-colonized explants occurred at a low level (12% to 19%), and those occurrences did not correlate well with differences in ergot alkaloid concentrations. Similarly, in preference tests pairing noncolonized SYE with either fungus-colonized explant, the fungus-colonized explant (though presumably lower quality) was selected 14% to 22% of the time. Beekman and Latty [30] discussed how *P. polycephalum* is not always rational and makes what appear to us to be irrational decisions occasionally, perhaps by selecting the first item that passes some threshold without processing all available information. The 12% to 22% of the time in which the apparently lower-quality option was selected in our study may be due to such intrinsic irrationality and/or sources of error inherent to our test plate setup. Such sources may include imprecise placement of the plasmodial inoculum relative to the treatment explants; potential differences in alkaloid content and residual nutrient concentrations among explant cores taken from different regions of the fungal culture plate; and differences in surface area in contact between the agar of the explant core and the agar surrounding the well in the water agar plate. Each of these sources of error would be expected to occur randomly among treatments and thus was unlikely to have biased the observed outcomes. Other factors that may have affected preference include circadian oscillations and changes in the availability of calcium and resources for protein synthesis in the fungal cultures used as explants, especially as those cultures (the sole source of nutrients for the plasmodia) aged and depleted nutrients in the medium. Each of these factors may affect the decision-making process of *P. polycephalum* plasmodia [39]. We attempted to minimize the effects of circadian oscillations by inoculating test plates and recording data at set times of day. Availability of calcium and other nutrients was presumed to be similar between cultures of the different fungal strains, but we cannot exclude the possibility that the genotypic changes that led to changes in ergot alkaloid concentration had pleiotropic effects.

Our results establish that plasmodia of *P. polycephalum* are sensitive to concentrations of ergot alkaloids in fungi available as food sources. Whether the foraging activities of *P. polycephalum* are affected by any of several other classes of mycotoxins remains to be investigated. In cases where purified chemicals are available, combining toxins with preferred nutrient sources would provide a more direct test of the effects of those chemicals. Our experiments were performed in darkness to limit the number of variables. Future experiments introducing light, as a perceived abiotic risk for *P. polycephalum* [40], also may be fruitful in understanding the degree to which plasmodia avoid certain metabolites.

## 4. Conclusions

Ergot alkaloids from the fungus *Aspergillus leporis* affected the foraging activity of the plasmodial slime mold, *Physarum polycephalum*. Concentrations of LAH and chanoclavine-I were correlated with lower preference by plasmodia, indicating they were repellent or deterrent to the slime mold. LAH appeared to be a stronger deterrent than chanoclavine-I. The results suggest an additional way by which ergot alkaloids may benefit their producing fungi through self-preservation and/or resource preservation.

## 5. Materials and Methods

### 5.1. Microorganisms and Culture Conditions

*Aspergillus leporis* States and M. Chr. strain NRRL 3216 was obtained from the USDA Agricultural Research Service Culture Collection (NRRL, Peoria, IL, USA). The lysergic acid amide-deficient, *easD* knockout strain of *A. leporis* was engineered and characterized previously [9]. The ergot alkaloid pathway in this strain had been mutated at the *easD* locus by CRISPR/Cas9-based technology because *easD* is represented functionally only once in the two lysergic acid amide synthesis clusters of the fungus [9,27]. A second functional copy of *easD* resides outside the lysergic acid amide clusters and accounts for low concentrations of lysergic acid amides in some cultures of the *easD* knockout strain, as well as for the accumulation of some fumigaclavine A [29]. Both strains of *A. leporis* were cultured on sucrose-yeast extract agar (SYE; per liter: 20 g sucrose, 10 g yeast extract, 1 g magnesium sulfate-heptahydrate, and 15 g agar) at 30 °C in an incubator with ambient oxygen.

*Physarum polycephalum* Schwein. was obtained from Carolina Biological Supply (Burlington, NC, USA) and maintained on 1.5% water agar supplemented with autoclaved rolled oats. Plasmodia were cultivated at room temperature (approximately 22 °C) in darkness. Tests of preference of plasmodia of *P. polycephalum* for explants of *A. leporis*, the *easD* knockout derivative, or noncolonized SYE were conducted at room temperature in darkness.

### 5.2. Analysis of Ergot Alkaloids

Ergot alkaloids of *A. leporis* and its *easD* knockout derivative were extracted from agar culture cores with an equal volume of methanol and analyzed by high-performance liquid chromatography (HPLC) with fluorescence detection by applying established methods described in detail previously [9,12,18,27,28,29]. The instrumentation was a Waters (Milford, MA, USA) Arc system set to monitor fluorescence at two sets of wavelengths. Lysergic acid derivatives were detected by excitation at 310 nm and monitoring emission at 410 nm. Chanoclavine-I and fumigaclavine A were analyzed with excitation at 272 nm and emission at 372 nm. The solid phase was a Phenomenex (Torrance, CA, USA) Prodigy C_18_ column (150 mm in length, 4.6-mm inside diameter, 5-µm particle size) and the mobile phase consisted of a binary, multilinear gradient from solution A (5% acetonitrile + 95% 50 mM aqueous ammonium acetate) to solution B (75% acetonitrile + 25% 50 mM aqueous ammonium acetate) over 30 min as described previously [28]. Chanoclavine-I, ergonovine, and fumigaclavine A were quantified by comparison of peak areas to standard curves prepared with authentic standards. Lysergic acid derivatives other than ergonovine (i.e., lysergic acid, lysergyl-alanine, ergine, and LAH) were quantified by comparing peak areas to a standard curve prepared from ergonovine (with which they share the same fluorophore), and thus quantities of those alkaloids must be considered as ‘relative to ergonovine’ as opposed to absolute. Sources of standards were as follows: chanoclavine-I (Alfarma, Prague, Czech Republic), ergonovine (Sigma-Aldrich, St. Louis, MO, USA), and fumigaclavine A (Alexis Biochemicals, San Diego, CA, USA).

### 5.3. Preference Tests

Tests of the effects of ergot alkaloids on the preference of plasmodia of *P. polycephalum* were conducted at room temperature in darkness on water agar (1.5%) plates containing only fungal culture explants (or noncolonized SYE explants in some experiments) as a source of nutrients. Two wells per test plate (positioned with the aid of the printed template in Figure S1) were cut by removing a cylinder (or core) of water agar by coring with the broader end of a 1000 µL disposable pipette tip (part number 13-611-123, Fisher Scientific, Pittsburgh, PA, USA). Explants of wild-type *A. leporis* or its *easD* knockout were prepared by similarly excising an approximately 400µL cylinder of colonized agar along with accompanying fungal material from an SYE culture with the broader end of a 1000 µL disposable pipette tip. The fungal/agar explant (containing the entire depth of the culture) was removed from the broader end of the pipette tip with the aid of a flame-sterilized dissecting needle and inserted, fungus side facing up, into a well in a 1.5% water agar plate. A small explant of inoculum from a plasmodium of *P. polycephalum* (from a culture on 1.5% water agar supplemented with rolled oats and less than 5 days old) was placed equidistant from the two fungal explants with a sterilized dissecting needle. The preference of plasmodia for either explant was observed 16 h after inoculation. Plasmodia were recorded as preferring wild-type *A. leporis* or the *easD* knockout mutant when they migrated toward and contacted only one of the explants. Plasmodia were scored as undecided when they went to and contacted both or neither of the explants. Preference for each explant (or undecided plasmodia) was calculated as a percentage of occurrences among the replicated pairings from that trial date.

Preference tests comparing the response of plasmodia of *P. polycephalum* to SYE colonized by wild-type *A. leporis* or its *easD* knockout derivative were conducted with eight trials for 6-day-old cultures and six trials for 13-day-old cultures. Each trial was conducted on a different date and contained multiple replicate plates. For trials with 6-day-old cultures, six of the trials contained 12 replicate plates each, one contained 15 replicate plates, and one trial contained 5 plates. Each of the six trials conducted with 13-day-old cultures included 12 replicate plates. Triplicate cores of fungus-colonized agar were removed from the same culture plate by the same approach described above for quantifying ergot alkaloids by HPLC (as described above in Section 5.2).

Tests of plasmodial preference for noncolonized SYE compared to SYE colonized by wild-type *A. leporis* or its *easD* knockout mutant were conducted and analyzed by the same methods, but with different numbers of trials and different numbers of replicate plates per trial. For wild-type *A. leporis* compared to SYE, six trials were conducted with 6-day-old cultures: one with 12 replicate plates and five with 6 replicate plates. Five trials were conducted to compare the *easD* knockout with SYE: one with 12 replicate plates and four with 6 replicate plates. For 13-day-old cultures of both strains, seven trials were conducted: one with 12 replicate plates, five with 6 replicate plates, and one with 4 plates. Preference was scored and calculated as a percentage value as described immediately above.

### 5.4. Statistical Analysis

All statistical analyses were conducted in JMP version 18 (SAS Institute, Cary, NC, USA). Concentrations of ergot alkaloids were compared by nonparametric Wilcoxon rank sum tests because variances were not homogeneous, as determined by Brown–Forsythe tests (*p* < 0.05). The lack of homogeneity in the variance of these data was expected and is affected by the large number of ‘not detected’ data points (scored as zero) in the alkaloids of the *easD* knockout. For each fungal culture included in a plasmodial preference trial, three cores (or explants) of the culture were analyzed by HPLC, and the mean of the three replicates was considered as representative of the concentration of each of the listed ergot alkaloids in that fungal strain on that trial date. The data shown in Figure 2 are overall means (with standard errors) of each of eight trial date means for 6-day-old cultures or from each of six trial date means for 13-day-old cultures.

Preference tests comparing the response of plasmodia of *P. polycephalum* to SYE colonized by wild-type *A. leporis* or its *easD* knockout derivative (or in some experiments to noncolonized SYE cores) were conducted in multiple, replicated trials as described above in Section 5.3. Within each trial date, the percentage of each explant chosen was calculated; the percentage of undecided plasmodia (those choosing neither nor, in rare cases, both explants) also was calculated. Data presented in Figure 4 and Figure 6 are the means (and standard errors) of the percentages calculated for each of the multiple trial dates. Data were checked for homogeneity of variances by Brown–Forsythe tests and passed (*p* > 0.05). Means were then compared by ANOVA, which indicated significant differences among treatment groups (*p* < 0.05). Means of individual treatments were then compared pairwise by Tukey’s honest significant difference test.

Correlations were tested by first calculating the difference in concentration of LAH (or chanoclavine-I, or total ergot alkaloids) separately for each trial date and correlating those differences with each of the potential preference outcomes observed on each of the respective trial dates. The difference in alkaloid concentration for a pairing was calculated by subtracting the concentration measured in the *easD* knockout culture from the concentration measured in the wild-type culture with which it was paired. Thus, when wild-type *A. leporis* contained more of an ergot alkaloid (or total ergot alkaloids) than the *easD* knockout strain, the difference was a positive value; when the *easD* knockout contained more of an alkaloid (or total ergot alkaloids) than the wild type, then the difference was a negative value.

## Data Availability

The original contributions presented in this study are included in the article and Supplementary Material. Further inquiries can be directed to the corresponding author.

## Supplementary Materials

The following supporting information can be downloaded at: https://www.mdpi.com/article/10.3390/toxins18070282/s1, Figure S1: Template for setting up paired preference tests of plasmodia of *Physarum polycephalum*; Table S1: Correlations of differences in ergot alkaloid concentration with foraging preference; Table S2: Ergot alkaloids whose concentration differences correlate with the concentration difference of LAH in wild-type *Aspergillus leporis*; File S1: Data used to create Figure 2, Figure 4, Figure 5 and Figure 6 and Tables S1 and S2 compiled in a Microsoft Excel workbook.

## Abbreviations

The following abbreviations are used in this manuscript:

LAH	Lysergic acid α-hydroxyethylamide
HPLC	High-performance liquid chromatography
*eas*	Ergot alkaloid synthesis (as applied to genes or gene clusters)
ANOVA	Analysis of variance
