# Supplementary material for: Ergot Alkaloids Affect Foraging Activity of the Slime Mold Physarum polycephalum
Source: Toxins (Basel). 2026 Jun 27;18(7):282. doi: 10.3390/toxins18070282 (PMC13417008; doi:10.3390/toxins18070282)
Supplement: Supplementary file 1 [file toxins-18-00282-s001.zip › figure S1.pdf]

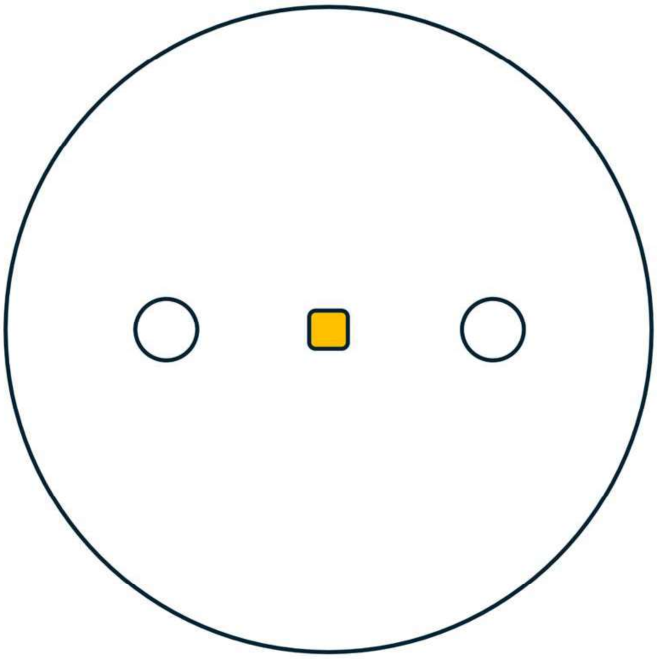

Figure S1. Template for setting up paired preference tests of plasmodia of *Physarum polycephalum*. The largest circle represents an 85-mm-diameter Petri dish. The two smaller circles represent 8-mm-diameter wells cored from the 1.5% water agar plate with the broader end of a 1000-μL pipette tip (part number 13-611-123, Fisher Scientific, Pittsburgh, PA, USA). Wells will be filled with explants of fungus-colonized (or in some cases noncolonized) agar medium. The center of each well is 21 mm from the nearest wall of the Petri dish and 21 mm from the center of the Petri dish. The yellow square in the center of the Petri dish represents inoculum from a plasmodium of *P. polycephalum*.
