# Supplementary material for: Ergot Alkaloids Affect Foraging Activity of the Slime Mold Physarum polycephalum
Source: Toxins (Basel). 2026 Jun 27;18(7):282. doi: 10.3390/toxins18070282 (PMC13417008; doi:10.3390/toxins18070282)
Supplement: Supplementary file 1 [file toxins-18-00282-s001.zip › Table S1.pdf]

**Table S1.** Correlations of differences in ergot alkaloid concentration with foraging preference

| **Choice** | **Alkaloid [difference]** [1] | $R^2$ | $P$ |
|---|---|---|---|
| *easD* knockout | lysergic acid | 0.07 | 0.35 |
| *easD* knockout | lysergyl-alanine | 0.14 | 0.19 |
| *easD* knockout | ergine | 0.18 | 0.13 |
| *easD* knockout | LAH | 0.44 | <0.01 |
| *easD* knockout | ergonovine | 0.18 | 0.14 |
| *easD* knockout | chanoclavine-I | 0.34 | 0.03 |
| *easD* knockout | fumigaclavine A | 0.03 | 0.52 |
| *easD* knockout | total ergot alkaloids | 0.47 | <0.01 |
| undecided | lysergic acid | 0.07 | 0.35 |
| undecided | lysergyl-alanine | 0.20 | 0.10 |
| undecided | ergine | 0.28 | 0.05 |
| undecided | LAH | 0.53 | <0.01 |
| undecided | ergonovine | 0.29 | 0.05 |
| undecided | chanoclavine-I | 0.21 | 0.10 |
| undecided | fumigaclavine A | 0.07 | 0.37 |
| undecided | total ergot alkaloids | 0.41 | 0.01 |
| wild type | lysergic acid | 0.01 | 0.78 |
| wild type | lysergyl-alanine | 0.13 | 0.21 |
| wild type | ergine | 0.21 | 0.11 |
| wild type | LAH | 0.15 | 0.16 |
| wild type | ergonovine | 0.22 | 0.09 |
| wild type | chanoclavine-I | 0.03 | 0.58 |
| wild type | fumigaclavine A | 0.08 | 0.33 |
| wild type | total ergot alkaloids | 0.01 | 0.72 |

[1] Alkaloid for which the concentration difference (concentration in wild-type *Aspergillus leporis* minus the concentration in the *easD* knockout mutant) is being correlated with the percent listed in the first column
