# Supplementary material for: Ergot Alkaloids Affect Foraging Activity of the Slime Mold Physarum polycephalum
Source: Toxins (Basel). 2026 Jun 27;18(7):282. doi: 10.3390/toxins18070282 (PMC13417008; doi:10.3390/toxins18070282)
Supplement: Supplementary file 1 [file toxins-18-00282-s001.zip › Table S2.pdf]

**Table S2.** Ergot alkaloids whose concentration differences correlate with the concentration difference of LAH in wild-type *Aspergillus leporis*

| **Alkaloid [difference]** | $R^2$ | $P$ |
|---|---|---|
| lysergic acid | 0.21 | 0.10 |
| lysergyl-alanine | 0.60 | <0.01 |
| ergine | 0.47 | <0.01 |
| ergonovine | 0.36 | 0.02 |
| chanoclavine-I | 0.03 | 0.56 |
| fumigaclavine A | 0.01 | 0.70 |
| total ergot alkaloids | 0.26 | 0.06 |
